# Supplementary material for: Patients experience of trauma adapted yoga as a health promoting activity in forensic psychiatric care
Source: Int J Qual Stud Health Well-being. 2025 May 28;20(1):2509803. doi: 10.1080/17482631.2025.2509803 (PMC12120864; doi:10.1080/17482631.2025.2509803)
Supplement: Clean copy ZQHW-S-2025-0109.R1.docx [file ZQHW_A_2509803_SM6051.docx]

**Patients experience of trauma adapted yoga as a health promoting activity in forensic psychiatric care**

1. Sofie Lundström^1,2*^, ORCID nr: 0000-0002-1530-1864
2. Nóra Kerekes^1,2^, ORCID nr: 0000-0002-8854-0399
3. Catrin Johansson^1,2^, ORCID nr: 0000-0003-2110-1823

^1^ Department of Health Sciences, University West, 461 86 Trollhättan, Sweden

^2^ Centre for Holistic Psychiatry Research (CHoPy), 431 60 Mölndal, Sweden

* Corresponding author: Sofie Lundström, Ph.D.

Institution for Health sciences,

University West, 461 86 Trollhättan, Sweden

email: [sofie.lundstrom@hv.se](mailto:sofie.lundstrom@hv.se)

**Abstract**

**Purpose:** Although the effects of yoga on psychiatric symptoms in psychiatric care are gaining increasing recognition, research within forensic psychiatric care, particularly on subjective experiences, remains limited. To address this gap, the present study aims to describe patients’ experience of trauma-adapted yoga (TAY) as a health-promoting activity in forensic psychiatric care. **Methods**: Twelve individual semi-structured interviews with patients in forensic psychiatric care were conducted and data were analyzed using inductive content analysis. **Findings:** The patients’ experience of TAY as a health-promoting activity was captured in an overarching theme, “To feel that one is connected to mind, body and soul in a way that can promote a sense of well-being in an uncertain existence”. This overarching theme contained four themes: *Strengthening the body*, *finding a calm place within oneself, something to do solely for oneself but together with others,* and *prerequisites for doing yoga*. **Conclusion:** The findings emphasize that TAY has the potential to be a valuable health-promoting activity for patients in forensic psychiatric care by facilitating their resources to manage stressors in everyday life. Therefore, it should be offered as a complement to other treatments to promote patients’ health and well-being in forensic psychiatric care.

**Keywords:** *forensic psychiatric care, health promotion, patients’ experiences, salutogenic theory, trauma-adapted yoga (TAY)*

**Introduction**

Yoga has been increasingly recognized as a beneficial activity for people in psychiatric care, with evidence to support its ability to alleviate symptoms associated with various mental health conditions. Yoga is broadly defined as a holistic mind-body discipline that combines physical postures, controlled breathing, and mindfulness practices. In trauma-informed adaptations, yoga emphasizes safety, choice, and interoceptive awareness, offering a non-verbal pathway to self-regulation and reconnection with the body (van der Kolk, 2014; Tibbitts et al., 2021). Numerous studies have demonstrated that yoga interventions can effectively reduce symptoms of anxiety and depression (Butterfield et al., 2017; Cramer, Lauche, et al., 2018; Cramer, Lauche, Langhorst, et al., 2013; Uebelacker & Broughton, 2018; Wu et al., 2023), trauma (Cramer, Anheyer, et al., 2018; Macy et al., 2018; van der Kolk et al., 2014) and stress (Penman et al., 2012). Yoga has also been shown to improve the quality of life of individuals with schizophrenia (Cramer, Lauche, Klose, et al., 2013). While these studies provide insight into the potential therapeutic effects of yoga, most have relied on quantitative outcome measures and have been conducted in general psychiatric or community settings. There is limited understanding of how individuals in more restrictive, high-security environments—such as forensic psychiatric care—experience yoga, particularly when it is adapted to account for trauma and mental illness.

Despite these documented benefits, accessing yoga in inpatient and forensic psychiatric care remains challenging because it is not typically included as part of conventional care. Yoga is categorized under the broader framework of complementary and alternative medicine (CAM), which encompasses care and treatment practices that fall outside established medical care systems (SOU 2019:28). Within CAM, certain methods are integrated alongside conventional medical treatment as complementary medicine (CM), whereas alternative medicine typically serves as a substitute for conventional approaches. When yoga is incorporated alongside conventional psychiatric treatments, it can be classified as CM. Research reveals that CAM methods are frequently used for mental health challenges, either in combination or as an alternative to conventional care (de Jonge et al., 2018; Hansen & Kristoffersen, 2016; Solomon & Adams, 2015). In Sweden, 62 percent of 489 healthcare units treating individuals with psychiatric symptoms reported using CAM for relief of symptoms such as anxiety, sleep disturbances and depression (Wemrell et al., 2020). However, CAM use in psychiatric care is a subject of ongoing debate. For example, Olsson et al. (2022) emphasized that there are diverging understandings about the use of CAM in psychiatric healthcare and skepticism toward CAM in specialist psychiatric care, citing concerns about its suitability and lack of integration into evidence-based practice. On the other hand, proponents have argued that CAM, including yoga, offers tangible benefits and enhances person-centered care. This perspective aligns with the principle that person-centered care is grounded in the recognition of each patient’s unique needs, preferences, life context, and values (Ekman, 2022). CAM frequently adopts a holistic approach that considers the individual as a whole—encompassing physical, psychological, and emotional dimensions—which aligns closely with the foundational principles of person-centered care.

Implementing yoga in forensic psychiatric contexts presents unique challenges. Forensic psychiatric care is a special setting for treating offenders with severe mental disorders who have been sentenced to care instead of prison (SFS 1991:1129). This form of care operates at the intersection of law, criminal justice, and clinical psychology. Patients in these settings often face complex life circumstances, including psychosocial, economic, environmental, and substance-related difficulties alongside their mental health disorders (Gordon & Lindqvist, 2007). The most prevalent diagnoses among patients in forensic psychiatric care are psychotic disorders, such as schizophrenia, although neurodevelopmental, personality, and substance use disorders have also been frequently observed, often co-occurring (Degl' Innocenti et al., 2014; Degl’ Innocenti et al., 2021). Furthermore, there is a high prevalence of post-traumatic stress disorder (PTSD) within this population (Bianchini et al., 2022). Patients’ experiences of forensic psychiatric care reveal a broad range of experiences that are associated with challenges in recovering (Askola et al., 2018; Marklund et al., 2020; Pollak et al., 2018; Zhong et al., 2019) such as feelings of hopelessness and worthlessness, and limited access to mental health interventions (Zhong et al., 2019). Patients emphasize the importance of meaningful activities, future planning (Pollak et al., 2018), being listened to, and being involved in their care (Askola et al., 2018; Marklund et al., 2020) to facilitate recovery from severe mental illness.

Yoga interventions have shown potential to address these challenges. Previous studies in forensic psychiatric settings have reported high attendance rates and benefits such as greater relaxation, reduced anxiety (Sistig et al., 2015), decreased stress, and an increased ability to describe feelings (Spinelli et al., 2020). For example, a 10-week study on trauma-adapted yoga (TAY) program significantly reduced anxiety, paranoid ideations, hostility, interpersonal sensitivity, overall psychological distress, and negative affect states among forensic psychiatric patients (Kerekes, 2024). Participants also reported reduced pain frequency and enhanced self-directedness. TAY is a Swedish adaptation of trauma-informed yoga, originally developed to address the needs of individuals with complex trauma and PTSD (Justice et al., 2018). TAY is specifically tailored for patients with trauma histories, including those diagnosed with severe mental illness, and accounts for the neurological, psychological, and physical challenges common in these populations. It serves as a complementary therapeutic practice that can support trauma recovery, reduce psychiatric symptoms, and enhance conventional treatment interventions (Kerekes, 2024). Rooted in trauma-informed principles, TAY modifies traditional yoga practices to ensure physical and emotional safety while promoting body awareness, emotional regulation, and rehabilitation. Sessions are structured to provide predictability, emphasize participant choice, use invitational language, and cultivate present-moment awareness (Kerekes, 2024; Justice et al., 2018). Although TAY aligns closely with other trauma-sensitive approaches, it is uniquely adapted for use in psychiatric institutions and also addresses the stress management needs of healthcare staff (Kerekes, 2024).

The need for health-promoting care that supports sustainable and equitable health is emphasized in Sweden’s Agenda 2030 action plan (Government Offices of Sweden, 2018). Health promotion involves empowering individuals to take control of their health and to influence the factors that contribute to well-being (WHO, 1986). The present study adopts a salutogenic perspective, which focuses on resources that promote health, and the processes that move individuals towards well-being (Antonovsky, 1979, 1987). Key concepts within the salutogenic theory include general resistance resources (GRR), specific resistance resources (SRRs), and sense of coherence (SOC). GRRs are the resources that facilitate an individual’s ability to cope with stressful situations in life. These resources can be diverse, encompassing material, physical, emotional, and cultural aspects, and can be found within the individual, their immediate environment, or society (Antonovsky, 1979, 1987). SRRs refer to resources used to address specific stressors in particular circumstances (Mittelmark et al., 2022). However, a prerequisite for being able to use available GRRs and SRRs in a way that promotes health is contingent upon their prior identification. SOC is a life orientation, a way of perceiving life as comprehensive, manageable, and meaningful (Antonovsky, 1987), reflecting confidence in one’s ability to use resources in a way that promotes health (Eriksson & Lindström, 2006). A strong SOC is associated with improved quality of life, better self-rated health, and, particularly, better mental health outcomes (Eriksson & Lindström, 2006; Eriksson & Lindström, 2007). Identifying and utilizing ones GRRs and SRRs can strengthen the development of SOC facilitating coping and promoting health (Suominen & Lindstrom, 2008). Given its emphasis on health resources, the salutogenic framework provides a suitable theoretical foundation for exploring yoga as a health promoting activity and its potential to promote resources for managing the stressors that individuals in forensic psychiatric care can face.

There is a growing body of research suggesting that yoga—particularly trauma-informed/adapted yoga—may support emotional regulation, stress coping, and recovery in vulnerable populations. However, research focusing on patients’ subjective experience of participating in yoga in mental health care remains limited and, to our knowledge, no prior study has examined patients’ experiences of TAY within forensic psychiatric care. The present study examines trauma-adapted yoga (TAY) as a form of CM in forensic psychiatric care, aiming to describe experiences of TAY as a health-promoting activity from the perspective of patients themselves.

**Method**

**Design**

This study is part of a larger project examining the effects of trauma-adapted yoga (TAY) on patients in forensic psychiatric care settings in Sweden. The project was conducted between 2022 and 2024 and involved seven forensic care units across the country. Healthcare workers from the participating forensic care unit were trained as TAY instructors to deliver the intervention. The patients participated on a voluntary basis in yoga at least once a week for a 10-week period. Each session lasted for approximately 45 minutes and was conducted in small groups of 8–10 participants or offered individually when necessary. The yoga sessions incorporated a range of trauma-informed techniques, including physical movements, balance exercises, adapted breathing methods, mindfulness practices and guided progressive muscle relaxation, tailored to the needs of the specific participant group. For a detailed description of the intervention, refer to Kerekes (2024). Since the present study aimed to explore patients’ experiences of participating in TAY practice in forensic psychiatric care settings, a qualitative descriptive approach was chosen. A qualitative approach is well-suited for deepening understanding, empowering individuals to share their stories, and ensuring that their voices are heard (Creswell & Creswell, 2018). Data were collected through qualitative interviews with patients who participated in the TAY program. The interviews focused on capturing participants’ perspectives of how yoga influenced their mental health and overall well-being. The study is reported in accordance with the Consolidated Criteria for Reporting Qualitative Research (COREQ) checklist (Tong et al., 2007).

**Participants**

Patients were recruited from seven forensic psychiatric care units participating in the larger project. Recruitment was facilitated by healthcare workers at each unit, who approached eligible patients and invited them to participate in the study. Patients received an information letter detailing the study’s aim and procedures, along with a short informational film created by the researchers who also conducted the interviews (SL, CJ). Inclusion criteria for participating in an interview were that the patients must have participated in yoga sessions on at least ten occasions, that they understand and speak Swedish, and that they have been admitted to forensic care for a minimum of two months. Out of 38 eligible patients, 12 ultimately agreed to participate in the interviews. The reason for not participating was unknown since the patient did not have to state a reason. The sample consisted of ten men and two women, from five different forensic psychiatric care units. Participants ranged from 27 to 54 years old (M=39) and had been admitted to the forensic psychiatric care for periods ranging from four months to six years. Of the 12 participants, only two had no prior yoga experience. The attendance rate at the yoga classes offered to the participants was high, with the majority having missed only a few of the 20 sessions offered during the 10-week period.

**Data collection**

Qualitative semi-structured interviews were employed for data collection conducted between January 2022 and January 2024. After patients consented to participate, an interview time was scheduled based on their preferences. Participants were offered the choice of conducting the interview digitally or at the forensic care unit, or via telephone. Six interviews were conducted digitally: five in person at the care units, and one by telephone per the patient’s request. The interviews lasted between 12 and 38 minutes. All interviews were conducted, audio-recorded and transcribed verbatim by two of the authors (SL and CJ), who are trained mental health nurses and PhD-level researchers. The interviewers and patients were not acquainted with each other prior to the interviews. All interviews, except one, were conducted with only the patient and the interviewer present; in one case, a staff member was also present due to regulations at the clinic. At the beginning of each interview, the researchers provided a recap of the study’s purpose and allowed participants to ask questions. Informed consent was then obtained before proceeding.

A semi-structured interview guide (Polit & Beck, 2021) was used to facilitate the interviews. The guide included open-ended questions, supplemented by follow-up questions, when necessary, to encourage in-depth responses. Each interview began with some introductory questions, such as how many times they participated in yoga sessions, whether they had prior experience of yoga, and their initial expectations of participating in yoga. The patients were then invited to share their reflections on their experiences of yoga, including aspects they enjoyed or disliked, its perceived effects on their physical and mental health, and any insights gained or ways they could apply yoga in the future. Throughout the interviews, the patients were encouraged to talk freely about their experiences with follow-up questions used as needed to clarify or expand upon their responses. During the interviews, the interviewers periodically summarized the patients’ descriptions to ensure accurate understanding of their experiences.

**Data analysis**

The interviews were analyzed using qualitative inductive content analysis (Graneheim & Lundman, 2004). First, the authors (SL and CJ) read the interviews several times to grasp an understanding of the content. Thereafter, they coded one interview together and then coded the rest of the interviews separately. In the process of coding, meaning units related to the research question were identified and condensed. These condensed meaning units were then abstracted and assigned codes that described their core content, resulting in a total of 282 codes. In the next step, the two authors collaboratively reviewed the codes together and reflected upon them based on similarities and differences, abstracted them, and sorted them into themes and subthemes. To preserve the patients’ voices, the analysis prioritized descriptions that remained close to the text rather than interpretations of the text. However, even if a text is analyzed at a manifest level in the coding process, the level of interpretation increases during the analysis when creating subthemes and themes (Graneheim et al., 2017). During the analysis, codes and themes were discussed and reflected upon by the authors, which led to some regrouping until a coherent pattern emerged that captured the essence of the patients’ experiences. Finally, a recurring overarching theme was identified, weaving through all themes and subthemes. An example of the analysis process, including meaning units, condensed meaning units, codes, subthemes, and themes, is presented in Table 1. The data analysis resulted in four themes and nine subthemes, and an overarching theme (see Table 2).

*Please insert Table 1 here.*

**Ethical considerations**

According to Swedish law (SFS 2003:460) concerning research on humans the study was approved by the Swedish Ethical Review Authority (Dnr 2021-01533). Informed written consent was given by the participants before the larger project started. Before the interviews, participants provided informed verbal consent and were informed orally and in writing about the purpose of the study. They were explicitly told that their participation was voluntary, that they could withdraw at any time without providing a reason, and that the interviews would be treated confidentially in accordance with ethical guidelines (World Medical Association, 2024). The patients were reassured that their participation, or decision to withdraw, would not affect their care in any way.

**Findings**

The patients’ experience of trauma-adapted yoga as a health promoting activity in forensic psychiatric care resulted in an overarching theme: *“To feel that one is connected to mind, body and soul in a way that can promote a sense of well-being in an uncertain existence/situation*”. This overall theme consisted of four themes: *“Strengthening the body”, Finding a calm place within oneself”, “Something to do solely for oneself but together with others”* and *“Prerequisites for doing yoga”.* These themes were based on the nine subthemes presented in Table 2.

*Please insert Table 2 here.*

**Strengthening the body**

The theme *Strengthening the body* includes the participants’ experiences of the physical effects of yoga and consists of two subthemes*: feeling more flexible* and *feeling strong and in control*. The first subtheme describes how yoga contributed to the participants’ experiences of reduced bodily stiffness, reduced pain and increased mobility. The second subtheme describes the participants’ experience of positive aspects of yoga on the body, such as increased muscle strength, endurance, body control, and balance.

**Feeling more flexible**

The participants shared that they had experienced stiffness in their bodies before they joined the yoga classes. They reported that yoga helped them feel less stiff and more flexible, which they greatly appreciated. Some participants admitted that they did not know much about yoga before joining the yoga classes but expected it to involve movement and stretching exercises, which aligned with their experiences. These exercises were perceived to contribute to an overall sense of increased mobility and physical ease.

*“I was very stiff in my body, very, very stiff after a lot of sitting, and I was even limping, but now it’s gone so it must be the yoga effect because I don’t do any other exercise...” (Interview 8)*

Participants emphasized the importance of learning how to stretch and relax through yoga. The chair-based exercises were particularly appreciated, as they could be easily performed in any seated setting. Some participants also noted a reduction in pain, particularly in the neck and legs, with some reporting that their pain had completely disappeared after starting yoga.

**Feeling strong and in control**

The participants reported feeling stronger and more resilient after engaging in yoga. They described benefits for their muscles and body control, along with an increased awareness of their bodies in a way that they had not experienced before, which they found positive.

*“I have more endurance now, I think because I’ve stretched my legs, I’ve got good endurance, better than before ... so I can go for walks and likewise.” (Interview 4)*

The participants described that their balance and body control improved after participating in yoga. The balance exercises were something that had worried them initially, but because the exercises were repetitive and gave them the opportunity to learn each movement properly, the control of their body got better for each time they practiced yoga. Improved posture was another commonly perceived benefit, with one participant noting that improved posture reduced bodily tension.

*“I think these balance exercises, in particular, take a bit of time, and then making sure you do them correctly – like the ones where you were lying down and tilting over with your knees. Getting the stretches right, finding the right muscles – each time, I got a little better at it. The first few times felt a bit unfamiliar though.” (Interview 9)*

Feeling physically stronger was also associated with having more energy, a positive outcome of yoga. This increased energy contributed to their ability to engage in everyday activities, including other physical activities.

*“Yes, I thought it would be good on a fairly broad basis, that you would become calmer and so... But what I’ve noticed the most is more energy.” (Interview 11)*

**Finding a calm place within oneself**

The theme *Finding a calm place within oneself* describes different aspects of yoga as a calming activity and consists of two sub-themes: *a general feeling of well-being* and *being able to manage thoughts, feelings and emotions,* which highlight two different dimensions of finding a calm place within oneself. The first subtheme presents a more general feeling of being able to relax in the moment and feeling reduced stress, while the second subtheme presents emotions and thoughts being affected on a deeper level, which increases the ability to manage negative thoughts and regulate overwhelming emotions.

**A general feeling of well-being**

The participants described that yoga had a calming effect and fostered a general sense of well-being and harmony. They noted that yoga helped them relax, especially during the yoga sessions, as it provided an opportunity to temporarily shut out the outside world. Some participants found that this feeling of calm and relaxation persisted beyond the yoga session, contributing to a sense of tranquility throughout the rest of the day.

*“Nervousness disappeared and I was calmer, and I’ve been searching for that, how can I be calmer and get through the day here...” (Interview 12)*

The participants described feeling less stressed as a result of participating in the yoga sessions. However, they also highlighted that it can be challenging to relax during the yoga session if they feel excessively stressed beforehand. The physical aspects of yoga, including balance exercises and stretching, were particularly appreciated by some, while others valued the final relaxation most. Breathing techniques were widely recognized as helpful and practical, with participants noting that they could apply these techniques in various contexts to manage stress and promote inner peace.

*There are some breathing exercises, just sitting relaxed and breathing. So, when I feel stressed, I use them – deep breaths and such – even when I’m not in a session.” (Interview 8)*

After completing the 10-week yoga program, some participants expressed that yoga not only provided immediate stress relief but also helped with long-term stress management.

*“It actually gave me peace in the longer term, because at the beginning it was tense and you didn’t know, but then when you had done it after these 10 weeks, you felt calm in your body, I wasn’t as tense, I kind of felt more at peace and I also felt that I wasn’t always stressing myself out about certain things that might have stressed me out before.” (Interview 7)*

The participants described that yoga also had a positive effect on their sleep. They used relaxation and breathing techniques to unwind before going to bed, which helped improve their sleep quality temporarily. The general perception of yoga was that it promoted a feeling of both physical and mental well-being, and the importance of having a balance between body and soul was highlighted.

**Being able to manage thoughts, feelings, and emotions**

The participants explained that yoga helped them manage their thoughts and emotions by providing an opportunity to focus and concentrate on something external, rather than being absorbed by their own intrusive or overwhelming thoughts and feelings. Participants stated that yoga gave them an opportunity to distract their thoughts and to get a break from their ruminations and inner voices.

*“It helps more than anything else I’ve tried before. Both mindfulness … and the more present you can be, the less bothered by voices and so on you get. So, it’s quite a positive thing that I feel.” (Interview 11)*

*“If you have a lot of thoughts and ruminations, anxiety and pondering ... there will be an interruption where you can’t do much else but follow these instructions and then you let go of these other things for that moment anyway. (Interview 1)*

The different movements during the yoga session helped the participants focus on their body instead of their thoughts. They described it as a way to be “mindful in their own body” (Interview 9). The participants appreciated the meditative part of yoga, where it was possible to focus inward. The participants expressed concerns about the future and acknowledged the tendency to become stuck in negative thought patterns. However, they described how yoga helped them lower their demands on themselves, reframe their thoughts, and feel more confident that they will be able to live a normal life. They described how yoga has taught them to be more in the present, which has made it easier for them to think in a more positive way about the future.

*“It’s worry about the future, worried about what will happen and what won’t happen, you don’t know you’re fantasizing ... But if you have yoga then you don’t focus on those thoughts anymore, then you have a little more harmony and yes ... relaxing thoughts.” (Interview 2)*

The participants noted that yoga has contributed to a better ability to manage their emotions. They explained that yoga helps them release tension in the body. The participants described how emotions such as sadness, joy, anger, and worry often take over and that it is difficult to regulate them and find an appropriate level of the emotions. They reflected on how yoga helps them become grounded in themselves and find emotional balance.

*“It has taught me, and is still teaching me, to master my emotions ... and to simply find a calm, new place within myself.” (Interview 3)*

**Something to do solely for oneself but together with others**

This theme consists of three subthemes: *a break from everyday life on one’s own terms, a complement to other activities or treatments,* and *being in togetherness* and highlighting different dimensions of yoga as an activity in forensic psychiatric care. The first subtheme addresses yoga as a welcome break in everyday life on the ward and that you could participate on your own terms. The second subtheme describes yoga in relation to other treatments and activities offered and how yoga can be seen as a complement to these. The third subtheme stresses that even if yoga is something you do for yourself, it is done together with others, which contributes to a feeling of togetherness.

**A break from everyday life on one’s own terms**

The participants felt that opportunities for activities are limited in forensic psychiatric care, and it is easy to end up just watching TV. Therefore, yoga was seen as a welcome break in everyday life routines, a fun activity to look forward to, and an opportunity to focus solely on oneself.

*“It made it easier to get through the spring... it felt like you had something to look forward to.” (Interview 7)*

*“It’s been nice, to get everything done, you know you can suffer sometimes because you don’t do enough, or that you don’t give ... to life, so it feels good, you feel that today I have done yoga, it is a good feeling.” (Interview 3)*

They appreciated that the yoga classes were accessible and not overly demanding, allowing everyone to participate according to their own abilities and ambitions. This was a new way of thinking for participants with high self-expectations or with a background in sports, as they were accustomed to focusing on performing. However, the approach that everyone could participate on their own terms and that the yoga was adapted based on each participant being valued over time. Additionally, the lack of compulsion to participate was seen as a positive aspect.

*“… I come from a sports background, so I tend to think that everyone should give their utmost in team sports and such ... But yoga isn’t really like that; you get to choose for yourself. Sometimes it can be a bit hard to accept that not everyone might be giving their absolute best, but are just going with the flow instead. (Interview 11)*

**A complement to other activities or treatments**

Yoga was perceived as calmer than the other activities and exercises offered to the participants in the forensic psychiatric unit and as providing an opportunity to exercise without having to join a sporting activity. Yoga was a soft and mindful alternative, which was appreciated. However, the participants believe that all activities are equally important and complement each other.

*“Yoga is made to make you feel good, while football ... you feel good from moving and it’s kind of fun and maybe you don’t think about things ... yoga feels more like some kind of magic, a formula for well-being and football is more a hobby.” (Interview 5)*

The participants also noted that yoga could be a complementary approach to pharmacological treatment, particularly when medication alone was perceived as insufficient.

*“The medicine dampens but doesn’t really take anything away, you just stay where you are. You don’t get better; you don’t get worse.” (Interview 11)*

*“Medicine doesn’t always help; it does help, but it’s not good for the body … but yoga is pretty good though.” (Interview 12)*

Most of the participants expressed a desire to continue practicing yoga in the future, whether in the forensic psychiatric unit, during leave, or after discharge. They emphasized that yoga’s benefits for both body and mind made it important to have opportunities to continue, either in arranged yoga groups together with others or via online yoga programs.

*“Yes, just continuing to attend the classes because they make me feel good in both body and soul. It has been very good and rewarding for me, and I plan to keep going.” (Interview 8)*

The fact that yoga was easy to perform and could be practiced in different places contributed to the participants’ desire to continue with yoga on their own.

**Being in togetherness**

The participants described that yoga as a group activity provided a sense of togetherness. It was enjoyable to do yoga together and being part of a group motivated them to participate. They also expressed that it was easier to learn yoga in a group because you could watch others do the movements. The feeling of togetherness was strengthened by the fact that the experience was shared by staff and patients.

*“Well, you have a little more harmony in the moment together, so it’s fun, you have something in common in the group that is going on.” (Interview 2)*

*“It [*group yoga*] gives off good feelings; you get to see others sometimes, how they move differently, and you notice, ‘Yeah, he’s doing it well.’ You get to observe other people, do it together, and see their reactions.” (Interview 6)*

The participants appreciated having both men and women in the group, as it reflected the typical composition of participants in settings such as gyms. However, one participant expressed anxiety about performing yoga in a group and found being in a group challenging. This difficulty was attributed to social anxiety experienced across various social contexts, rather than being specific to the yoga sessions.

**Prerequisites for doing yoga**

This theme consists of descriptions of various aspects that the participants see as prerequisites for enabling participation in yoga. The first subtheme, *to have the courage to try,* shows that it is important to overcome ones and others’ doubt about yoga and the second subtheme, *to be in a calm and permissive environment*, emphasizes that the environment has an impact on the participants experience of yoga.

**To have the courage to try**

Some of the participants had prior experience with yoga and knew roughly what to expect, while it was entirely new for others, which evoked feelings of hesitation and nervousness. However, they emphasized that it is important to dare to try and not be influenced by preconceptions that it would be difficult, overly spiritual, or boring. Several participants noted that they had a different idea of ​​yoga once they tried it.

*“Yes, you can try, you can’t think that everything is boring and so on until you have tried it ... and see how ... And I was a little surprised that it worked so well with this calmness and all that.” (Interview 12)*

The participants also noted prejudices about yoga being better suited for women and as unmanly. To address this, they emphasized the need for tailored information specifically designed to engage men, encouraging them to overcome these biases and try yoga. Overall, the importance of how yoga is presented was highlighted as crucial for counteracting such stereotypes. Participants suggested that information about yoga should be communicated in a simple and accessible manner, with patients serving as ambassadors to promote its benefits.

*“I tried to get some friends from the ward to join and tried to explain in my own way what I thought yoga was ... so maybe it’s presented a bit poorly for those who haven’t had any contact with it before ...” (Interview 9)*

*“Yes, it was good, I can recommend it to others and so on … and I’ve done that to people in here.” (Interview 10)*

**To be in a calm and permissive environment**

The participants emphasized the importance of a calm environment, which helped them concentrate on the movements, relax, and focus on themselves. They described several factors that contributed to creating such an environment, including shutting out outside sounds, using dim and cozy lighting, playing quiet music, providing something soft to lie on, and offering blankets to stay warm.

*“They made it a little cozier during the yoga; not candles but almost, those battery candles … so that it still felt like … you become calm in there.” (Interview 7)*

The role of the instructors was also highlighted as crucial in creating a calm atmosphere in the yoga room. Participants felt it was important for instructors to remain calm, provide clear instructions for the various exercises, and allow adequate time for participants to learn the movements at their own pace. Some participants noted that they initially found certain movements difficult, making it essential to feel supported rather than rushed. While most participants appreciated the sessions, some found them a little long and expressed difficulty maintaining concentration throughout. Some participants proposed ending each session with the option of a cup of tea to further enrich the atmosphere and provide a comforting conclusion to the practice.

**To feel that one is connected to mind, body and soul in a way that can promote a sense of well-being in an uncertain existence**

Overall, the patients’ experience of TAY as a health-promoting activity in forensic psychiatric care initiated a sense of connection between mind, body and soul in a way that can promote a sense of well-being in an uncertain existence. The participants perceived yoga as a holistic practice that integrated physical, mental and emotional aspects into a harmonious whole, blending depth with an awareness of the present. Through this practice, they developed a balance between physical strength and mental resilience while cultivating a sense of well-being. This delicate interplay of strength and stillness held particular significance within the complex and often challenging reality of receiving care in a forensic psychiatric setting. Yoga provided participants with resources to manage these challenges, promoting health and enhancing their ability to navigate their circumstances.

**Discussion**

Several studies have explored the therapeutic effects of yoga (Butterfield et al., 2017; Macy et al., 2018; Penman et al., 2012; Uebelacker & Broughton, 2018; van der Kolk et al., 2014; Wu et al., 2023). Notably, in a randomized clinical trial, Zaccari et al. (2023) compared a specific yoga intervention—Trauma Center Trauma-Sensitive Yoga (TCTSY)—with cognitive behavioral therapy (CBT) in women with PTSD related to military sexual trauma. Both interventions were found to be effective, yet TCTSY demonstrated higher participant satisfaction and lower dropout rates, suggesting that it may offer greater acceptability and feasibility for certain populations.

As part of a broader research project led by Kerekes (2024), of which the present study is a part, a quasi-experimental design was used to examine Trauma Adapted Yoga (TAY) in comparison with treatment as usual (TAU) within a forensic psychiatric setting. However, no such comparison is made in the current study, which instead focuses solely on participants' experiences of the yoga intervention. The absence of comparative analysis in the present study represents an important area for further research, particularly considering findings such as those by Zaccari et al. (2023). O'Shea et al. (2022) also emphasize the need for more integrative approaches that combine yoga-based methods with established, evidence-based psychological treatments, underscoring the importance of building a more cohesive and comprehensive treatment framework.

Nevertheless, this study contributes uniquely by exploring the experiences of patients participating in yoga within forensic psychiatric care. There is a paucity of research examining such subjective experiences, and addressing this gap can deepen our understanding of the role of yoga in complex clinical settings. To this end, the discussion situates the present findings within the context of existing research and draws on the salutogenic theory to interpret the results.

The participants experienced that yoga strengthens the body in various ways, which is crucial for promoting overall health in individuals within forensic psychiatric care. Physical activity is well-documented to enhance mental health and well-being (Vella et al., 2023), alleviate symptoms of anxiety and depression (Singh et al., 2023), and reduce cardiometabolic risk factors, functional disability, and psychiatric symptoms in individuals with schizophrenia (Firth et al., 2015). Engaging in physical activity is considered a preventive health-oriented approach, serving as an important GRR for health promotion (Antonovsky, 1987). However, it is essential that the activity is perceived as meaningful and contributes to a general sense of well-being (Lundström et al., 2017). Thus, yoga offers an alternative for those who may not feel comfortable with other forms of physical activity but still want to be physically active in some way. The findings also reveal experiences of reduced pain following yoga participation, which is consistent with previous measurement on the same study population (Kerekes, 2024).

Beyond physical benefits, yoga was described as facilitating a sense of calm and providing respite from the uncertainty of forensic psychiatric care, where the lack of clarity regarding the duration of admission often leads to frustration and distress (Marklund et al., 2020). Our findings show that the yoga session provides a temporary escape from this uncertainty, allowing participants to shut out external stressors and focus inward. This aligns with the salutogenic framework, where GRRs and SRRs can help individuals cope with challenges in life (Antonovsky, 1979; Mittelmark et al., 2022). Yoga sessions in forensic psychiatric care appear to enhance participants’ SRR by equipping them with tools to manage the inherent uncertainty of their circumstances. Furthermore, our finding revealed that yoga enhances stress management in both the short and long term. This aligns with Mandlik et al. (2024) systematic review, which found that a single yoga session significantly reduces acute stress reactivity in adults. Yoga’s potential to alleviate stress has been well-documented in the general population (Pascoe & Bauer, 2015; Penman et al., 2012; Wang & Szabo, 2020) and in correctional settings (Bilderbeck et al., 2013; Kerekes et al., 2017). While research on yoga in forensic psychiatric care is limited, earlier studies suggest that yoga may reduce stress in these contexts (Sistig et al., 2015; Spinelli et al., 2020).

While well-being was described in broad terms, the relationship to thoughts, feelings, and emotions was articulated more precisely particularly in managing and regulating negative thoughts and emotions that arose in different situations, such as worries, anxiety, annoyance and anger. Emotional regulation has been identified as a key benefit of yoga (Büssing et al., 2012). These findings are consistent with Kerekes (2024), who reported significant improvements in self-directedness among participants in the same population. Enhanced self-directedness, reflecting greater self-control and adaptability, can strengthen a person’s SOC (Antonovsky, 1979, 1987) and contribute to better stress management.

Using yoga as a strategy to manage negative thoughts, feelings and emotions was highlighted as important reflection. Participants also described an awareness of various strategies learned during yoga sessions, such as breathing techniques, body awareness, and relaxation, which they could apply in daily life to promote health and manage stress. By identifying and utilizing these strategies as GRRs, participants gained a sense of agency and a perception of control over their situations, fostering positive experiences and resilience in the face of future challenges.

Another notable finding was that yoga sessions provided a break from intrusive thoughts and inner voices. This aligns with Yin et al. (2024), who found that yoga had a moderate strength effect on positive symptoms (such as auditory hallucinations) in the short term, and a small strength effect in the long term for patients with schizophrenia. However, those studies were conducted in inpatient and outpatient psychiatric care settings, unlike the forensic psychiatric care context of the present study. Sathyanarayanan et al. (2019) also reported improvements in both negative and positive symptoms of schizophrenia, including emotional withdrawal and unusual thought content, through yoga interventions. Notably, none of the reviewed studies showed a worsening of psychotic symptoms. While most studies in this review included patients with mild to moderate psychopathology in general psychiatric settings, the present study extends these findings to the forensic psychiatric context, highlighting yoga’s feasibility and potential as a complementary treatment. Furthermore, several studies have shown that social and cognitive function and quality of life improved after participating in yoga (Sathyanarayanan et al., 2019; Vancampfort et al., 2021). This aligns with the findings of the present study, where participants noted yoga’s ability to enhance focus, foster present-moment awareness, and encourage optimism about the future, all of which contribute to an improved sense of well-being.

An important finding was that participants experienced yoga as an activity they could engage in solely for themselves, free from demands and external expectations. They appreciated that the yoga sessions were easy to perform and adaptable to one’s own capacity, allowing participants to feel capable and in control. Although participants spoke about yoga in general terms, this aligns with the trauma-adapted nature of the specific yoga practiced (TAY), designed to address the specific needs of individuals with psychiatric conditions and trauma experiences (Kerekes, 2024). Key aspects of TAY include creating a safe environment, maintaining a predictable structure, fostering present-moment awareness, and empowering participants to choose their level of engagement (Kerekes, 2024). These elements likely contributed to participants’ positive experiences. The feeling of doing something meaningful for themselves and becoming more involved in their care have been identified as important salutogenic factors for patients in forensic psychiatric care (Askola et al., 2018; Marklund et al., 2020). Being involved in decisions about oneself is a significant life experience that fosters a sense of meaningfulness. A structured environment enhances the feeling of consistency, and the perception that demands align with one’s resources is vital for manageability (Idan et al., 2022). Thus, the elements of TAY may contribute to strengthening the SOC for people cared for in forensic psychiatric care.

An important experience of conducting yoga in groups is the sense of togetherness that arises from shared experiences. However, participating in a group can also be challenging due to social anxiety. This aligns with findings from a study on yoga interventions for individuals with long-term mental health conditions (Snaith et al., 2020), which highlighted the importance of practicing yoga with peers for feeling comfortable, while also noting that anxiety could hinder participation. The same study emphasized the significance of a supportive relationship with the yoga teacher. This finding was consistent with our study, where the instructor’s role in fostering a supportive and permissive atmosphere was highlighted. Social and supportive relationships are crucial GRRs for managing life’s challenges (Antonovsky, 1979). It can be inferred that group yoga sessions may contribute to supportive social relations for patients in forensic psychiatric care by facilitating interactions beyond their patient identity and typical ward environment. However, the findings of the present study and of Snaith et al. (2020) indicate that a calm, private, permissive, and cozy environment is essential for participants to feel safe in a group setting. Additionally, providing tailored information to prepare participants for yoga appears to be important for lowering their expectations and encouraging participation based on their individual conditions.

The overarching theme identified in this study – *to feel that one is connected to mind, body and soul in a way that can promote a sense of well-being in an uncertain existence* – captures the latent meaning of the participants’ experiences of yoga as a health-promoting activity. Through the participants’ stories, it emerges that yoga could provide ease for a troubled mind, enhanced bodily awareness and strength, and creating meaning in everyday life. These aspects have the potential to promote overall health for people in forensic psychiatric care and can be seen as important resources for navigating the challenges of forensic psychiatric care, both in the present and in future contexts.

**Methodological considerations**

Qualitative content analysis enabled us to identify variations of the participants’ experiences of participating in yoga in forensic psychiatric care. To grasp the content of the participants’ utterances, the analysis was initially conducted at a manifest level with descriptions close to the interview text. However, there is always a degree of interpretation, even in manifest analysis depending on the level of abstraction of the text (Graneheim et al., 2017). Thus, the analysis is both descriptive and interpretive and has moved from a concrete level to a more abstracted level to be able to deepen the analysis and create suitable subthemes and themes. These phases of de-contextualization and re-contextualization in the analytic process strengthen trustworthiness (Lindgren et al., 2020). However, there may be a risk that the content is analyzed in an overly mechanical and distanced manner, thereby losing the meaning of the phenomenon itself (Dahlberg et al., 2024). To describe the meaning of the subjective experience, the authors have strived to preserve the unique experience of the participants by returning to the interview and interpreting them within their context. The analysis has been constantly discussed between the authors to reach consensus of the content, which can be considered as a strength of the study. The authors (SL and CJ) have experience of psychiatric care as mental health nurses, which probably affected the interpretation of the participants’ utterance and made it possible to see the content in the correct context.

The data were based on twelve interviews, which can be considered a limitation of the study. However, the participants gave many descriptions of their different experiences even if the interviews varied in richness and depth. Quotations were used to illustrate the participants’ own voices, which enhances credibility. The participants came from five different forensic psychiatric care units and varied in gender, age, and in time spent on the ward. A limitation was that only two women participated in the interviews, which means that experiences from a women’s perspective are scarce. However, this is representative for the context since more men than women are admitted to forensic psychiatric care. Only patients who spoke and understood Swedish were included in the interviews which can be considered as a weakness of the study. However, all patients were welcome to join the yoga sessions even if they did not participate in the interviews. Another limitation of the present study is the lack of information regarding the specific psychiatric diagnoses the participants were living with. However, in the larger project (Kerekes, 2024), it was found that participants experienced a range of different psychiatric conditions, with schizophrenia and substance-use-related syndrome being the most frequently occurring, which aligns with Degl' Innocenti et al. (2014) and Degl’ Innocenti et al. (2021).We can only assume that the distribution may be similar among the participants in the present study, although this cannot be determined with certainty. The intervention was conducted on a voluntary basis and followed a trauma-informed pedagogical approach, which emphasizes predictability, choice, and emotional and physical safety. Participants were given the option to engage in exercises to the extent they felt comfortable, including the ability to choose between different versions of each task and to be informed about the duration and structure of the sessions. As a result, not all patients participated in every part of the yoga practice. These variations in individual participation may have influenced their subjective experiences and, consequently, the result. Nevertheless, the diversity of participation reflects the core principle of self-agency in trauma-informed care, and the collective experiences of the participants offer valuable insight into how TAY is perceived and experienced in forensic psychiatric settings.

Healthcare workers recruited participants to the interviews, which can be seen as a weakness as the researcher cannot control who is asked to participate. This issue was discussed with the involved healthcare workers and the importance of asking everyone who met the inclusion criteria, and not only the one that was found suitable, was emphasized. Another potential sampling bias was that 26 out of 38 eligible participants chose not to participate, and the one who chose to participate may have been the ones who had a predominantly positive attitude towards yoga.

People cared for in forensic psychiatric care can be seen as a vulnerable group to engage in research because the care are involuntary and that they have a serious mental illness. However, the Declaration of Helsinki (World Medical Association, 2024) emphasizes that people who are underrepresented in research should be given opportunities to participate in research and ethical issues were constantly considered to avoid discomfort for the participants. It is important to highlight their experiences to examine whether yoga, as a complement to other care, is perceived to have an impact on health. The interviews were conducted with awareness and respect for the patients’ situation and various conditions. During the interviews, considerable emphasis was placed on creating a relaxed atmosphere so that the patients would feel safe talking about their experiences. Questions about how they felt yoga affected their mental health can bring up thoughts and feelings that feel uncomfortable. Therefore, the researchers were keen to be sensitive to how the participants felt about talking about their experiences and adapted the interview accordingly. Before the interview, it was also communicated that it was optional what and how much they wanted to say and that they had the option to cancel the interview at any time. Some interviews were conducted face to face and some digitally, which has pros and cons. On one hand, it can be more difficult to read body language and see, for example, emotional reactions during digital interviews. On the other hand, digital interviews can be experienced as less emotionally demanding because meetings via a camera can provide a distance that may make it easier to talk openly. The patients could have restrictions on using the computer alone, which made it challenging to ensure that they felt they could talk openly even though staff were present. Such issues were solved by having the staff wear headphones with, for example, music.

As with other qualitative research, the results cannot be generalized to a broader context; it is up to the reader to determine whether the findings may be transferable (Graneheim & Lundman, 2004). However, we believe the results can be transferable to similar contexts. As always, it is important to consider individual differences, which can be addressed by adopting a person-centered approach to care. This involves tailoring interventions to the patients’ circumstances, including their interests, suitability, and willingness to participate along with tailored information about TAY.

**Conclusion**

This study explores TAY as a CM intervention within forensic psychiatric care, emphasizing its role as a health-promoting activity for patients in this complex and uncertain context. The findings indicate that patients perceived TAY not merely as a physical activity, but as a holistic tool for enhancing well-being on physical, emotional, and mental levels. In alignment with the salutogenic theory, TAY provided participants with GRRs and SRRs that are important resources they could utilize to manage stressors of everyday life in forensic psychiatric settings. The structured and trauma-sensitive approach of TAY enabled them to identify and strengthen their internal and external resources, with the potential to strengthen their SOC, particularly through the domains of meaningfulness, and fostering a greater sense of control and adaptability in challenging circumstances. This focus on self-care and empowerment has the potential to instill optimism and hope, which are essential for navigating the uncertainties of forensic psychiatric care. Based on these findings, TAY appears to be a valuable addition to existing treatment modalities within forensic psychiatric care. By addressing the physical, mental, and emotional needs of patients, it offers a multidimensional approach to health promotion.

**Practical Implications and Future Research**

Implementing TAY in forensic psychiatric care presents a unique opportunity to enhance patient well-being and promote innovative care practices tailored to this vulnerable population. TAY’s adaptability and focus on trauma-sensitive framework make it particularly relevant for addressing the complex needs of patients. TAY is not a generic form of yoga, but a structured and highly adapted approach that requires instructors (healthcare professionals) to undergo specific training. This ensures that instructors are equipped with the necessary skills to deliver TAY safely and effectively. In this context, healthcare professionals across various disciplines within forensic psychiatry could benefit from obtaining TAY instructor certification. By doing so, they can expand the scope of therapeutic interventions available to patients and integrate meaningful and health-promoting activities into routine care.

Despite the promising findings of this and previous studies on TAY, further research is needed to solidify the scientific foundation of TAY in forensic psychiatric and psychiatric care. This is the first study to explore patients’ experiences of TAY in specific context, highlighting the need for future studies to examine its impact more comprehensively. Research should focus on both the measurable effects of yoga such as improvements in mental health, emotional regulation, and physical well-being, as well as the subjective experiences of participants, which can provide deeper insights into the intervention’s effectiveness. Additionally, the fact that the current study involved only two women as participants raises important questions about potential gender-specific experiences and outcomes of TAY. Future research should explore how gender and other demographic factors may influence participants’ engagement with and benefits from TAY. Moreover, studies should investigate the implementation of TAY in diverse psychiatric care settings, including outpatient, inpatient, and forensic environments, to better understand its applicability and efficacy across different populations and contexts.

**Acknowledgement**

We extend our heartfelt gratitude to the patients and healthcare workers who generously contributed their time and insights to this study. We also wish to express our sincere appreciation to Josefin Wikström for her invaluable role in training healthcare professionals in TAY, which made this research possible.

**Disclosure statement**

The authors report there are no competing interests to declare.

**Data availability statement**

The data supporting the findings of this study are not publicly available due to ethical and confidentiality considerations.

**Author contributions statement**

SL and CJ were responsible for data collection, analysis, and drafting the manuscript, including the initial sketches and full writing process. NK, as the project leader, provided overall guidance, critically reviewed the manuscript, and contributed to refining its content and structure. All authors approved the final version of the manuscript and agree to be accountable for all aspects of the work.

**Funding**

The author(s) reported there is no funding associated with the work featured in this article.

**References**

Antonovsky, A. (1979). *Health, stress and coping: new perspectives on mental and physical well-being*. Jossey-Bass.

Antonovsky, A. (1987). *Unraveling the mystery of health: how people manage stress and stay well*. Jossey-Bass.

Askola, R., Nikkonen, M., Paavilainen, E., Soininen, P., Putkonen, H., & Louheranta, O. (2018). Forensic Psychiatric Patients' Perspectives on Their Care: A Narrative View. *Perspectives in Psychiatric Care*, *54*(1), 64-73. <https://doi.org/10.1111/ppc.12201>

Bianchini, V., Paoletti, G., Ortenzi, R., Lagrotteria, B., Roncone, R., Cofini, V., & Nicolò, G. (2022). The Prevalence of PTSD in a Forensic Psychiatric Setting: The Impact of Traumatic Lifetime Experiences. *Frontiers in Psychiatry*, *13*. <https://doi.org/10.3389/fpsyt.2022.843730>

Bilderbeck, A. C., Farias, M., Brazil, I. A., Jakobowitz, S., & Wikholm, C. (2013). Participation in a 10-week course of yoga improves behavioural control and decreases psychological distress in a prison population. *Journal of Psychiatric Research*, *47*(10), 1438-1445. <https://doi.org/https://doi.org/10.1016/j.jpsychires.2013.06.014>

Butterfield, N., Schultz, T., Rasmussen, P., & Proeve, M. (2017). Yoga and mindfulness for anxiety and depression and the role of mental health professionals: a literature review. *The Journal of Mental Health Training, Education and Practice*, *12*(1), 44-54. <https://doi.org/10.1108/JMHTEP-01-2016-0002>

Büssing, A., Michalsen, A., Khalsa, S. B., Telles, S., & Sherman, K. J. (2012). Effects of yoga on mental and physical health: a short summary of reviews. *Evidence-Based Complementary and Alternative Medicine*, *2012*, 165410. <https://doi.org/10.1155/2012/165410>

Cramer, H., Anheyer, D., Saha, F. J., & Dobos, G. (2018). Yoga for posttraumatic stress disorder - a systematic review and meta-analysis. *BMC Psychiatry*, *18*(1), 72. <https://doi.org/10.1186/s12888-018-1650-x>

Cramer, H., Lauche, R., Anheyer, D., Pilkington, K., de Manincor, M., Dobos, G., & Ward, L. (2018). Yoga for anxiety: A systematic review and meta-analysis of randomized controlled trials. *Depression and Anxiety*, *35*(9), 830-843. <https://doi.org/10.1002/da.22762>

Cramer, H., Lauche, R., Klose, P., Langhorst, J., & Dobos, G. (2013). Yoga for schizophrenia: a systematic review and meta-analysis. *BMC Psychiatry*, *13*, 32. <https://doi.org/10.1186/1471-244x-13-32>

Cramer, H., Lauche, R., Langhorst, J., & Dobos, G. (2013). Yoga for depression: a systematic review and meta-analysis. *Depression and Anxiety*, *30*(11), 1068-1083. <https://doi.org/10.1002/da.22166>

Creswell, J. W., & Creswell, J. D. (2018). *Research design : qualitative, quantitative, and mixed methods approaches*. SAGE.

Dahlberg, H., Dahlberg, K., & Holmberg, C. (2024). The search for meaning in health care inquiries: introducing qualitative meaning analysis. *International Journal of Qualitative Studies on Health and Well-Being*, *19*(1). <https://doi.org/10.1080/17482631.2024.2382809>

de Jonge, P., Wardenaar, K. J., Hoenders, H. R., Evans-Lacko, S., Kovess-Masfety, V., Aguilar-Gaxiola, S., Al-Hamzawi, A., Alonso, J., Andrade, L. H., Benjet, C., Bromet, E. J., Bruffaerts, R., Bunting, B., Caldas-de-Almeida, J. M., Dinolova, R. V., Florescu, S., de Girolamo, G., Gureje, O., Haro, J. M., Hu, C., Huang, Y., Karam, E. G., Karam, G., Lee, S., Lépine, J. P., Levinson, D., Makanjuola, V., Navarro-Mateu, F., Pennell, B. E., Posada-Villa, J., Scott, K., Tachimori, H., Williams, D., Wojtyniak, B., Kessler, R. C., & Thornicroft, G. (2018). Complementary and alternative medicine contacts by persons with mental disorders in 25 countries: results from the World Mental Health Surveys. *Epidemiol Psychiatr Sci*, *27*(6), 552-567. <https://doi.org/10.1017/s2045796017000774>

Degl' Innocenti, A., Hassing, L. B., Lindqvist, A. S., Andersson, H., Eriksson, L., Hanson, F. H., Möller, N., Nilsson, T., Hofvander, B., & Anckarsäter, H. (2014). First report from the Swedish National Forensic Psychiatric Register (SNFPR). *International Journal of Law and Psychiatry*, *37*(3), 231-237. <https://doi.org/10.1016/j.ijlp.2013.11.013>

Degl’ Innocenti, A., Alexiou, E., Andiné, P., Striskaite, J., & Nilsson, T. (2021). A register-based comparison study of Swedish patients in forensic psychiatric care 2010 and 2018. *International Journal of Law and Psychiatry*, *77*, 101715. <https://doi.org/https://doi.org/10.1016/j.ijlp.2021.101715>

Ekman I. (2022). Practising the ethics of person-centred care balancing ethical conviction and moral obligations. *Nursing philosophy : an international journal for healthcare professionals*, *23*(3), e12382. <https://doi.org/10.1111/nup.12382>

Eriksson, M., & Lindström, B. (2006). Antonovsky's sense of coherence scale and the relation with health: a systematic review. *Journal of Epidemiology and Community Health*, *60*(5), 376-381. <https://doi.org/10.1136/jech.2005.041616>

Eriksson, M., & Lindström, B. (2007). Antonovsky's sense of coherence scale and its relation with quality of life: a systematic review [Article]. *Journal of Epidemiology and Community Health*, *61*(11), 938-944. <https://doi.org/10.1136/jech.2006.056028>

Firth, J., Cotter, J., Elliott, R., French, P., & Yung, A. R. (2015). A systematic review and meta-analysis of exercise interventions in schizophrenia patients [Article]. *Psychological Medicine*, *45*(7), 1343-1361. <https://doi.org/10.1017/S0033291714003110>

Gordon, H., & Lindqvist, P. (2007). Forensic psychiatry in Europe. *Psychiatric Bulletin*, *31*(11), 421-424. <https://doi.org/10.1192/pb.bp.107.014803>

Government Offices of Sweden. (2018). *Handlingsplan Agenda 2030. (Action plan for Agenda 2030).* <https://www.regeringen.se/rapporter/2018/06/handlingsplan-agenda-2030/>

Graneheim, U. H., Lindgren, B. M., & Lundman, B. (2017). Methodological challenges in qualitative content analysis: A discussion paper. *Nurse Education Today*, *56*, 29-34. <https://doi.org/10.1016/j.nedt.2017.06.002>

Graneheim, U. H., & Lundman, B. (2004). Qualitative content analysis in nursing research: concepts, procedures and measures to achieve trustworthiness. *Nurse Education Today*, *24* (2), 105-112. <https://doi.org/10.1016/j.nedt.2003.10.001>

Hansen, A. H., & Kristoffersen, A. E. (2016). The use of CAM providers and psychiatric outpatient services in people with anxiety/depression: a cross-sectional survey. *BMC Complementary and Alternative Medicine*, *16*(1), 461. <https://doi.org/10.1186/s12906-016-1446-9>

Idan, O., Eriksson, M., & Al-Yagon, M. (2022). Generalized Resistance Resources in the Salutogenic Model of Health. In M. Mittelmark, G. Bauer, & L. e. a. Vaandrager (Eds.), *The Handbook of Salutogenesis* (2nd ed., pp. 93-106). Springer. <https://doi.org/10.1007/978-3-030-79515-3_12>

Justice, L., Brems, C., & Ehlers, K. (2018). Bridging Body and Mind: Considerations for Trauma-Informed Yoga. *International Journal of Yoga Therapy*, *28*(1), 39-50. <https://doi.org/10.17761/2018-00017r2>

Kerekes, N. (2024). Exploring the impact of trauma-adapted yoga in forensic psychiatry. *Psychiatry Research*, *335*, 115879. <https://doi.org/10.1016/j.psychres.2024.115879>

Kerekes, N., Fielding, C., & Apelqvist, S. (2017). Yoga in correctional settings: A randomized controlled study. *Frontiers in Psychiatry*, *8*. <https://doi.org/10.3389/fpsyt.2017.00204>

Lindgren, B.-M., Lundman, B., & Graneheim, U. H. (2020). Abstraction and interpretation during the qualitative content analysis process. *International Journal of Nursing Studies*, *108*, 103632. <https://doi.org/https://doi.org/10.1016/j.ijnurstu.2020.103632>

Lundström, S., Ahlström, B. H., Jormfeldt, H., Eriksson, H., & Skärsäter, I. (2017). The Meaning of the Lived Experience of Lifestyle Changes for People with Severe Mental Illness. *Issues in Mental Health Nursing*, *38*(9), 717-725. <https://doi.org/10.1080/01612840.2017.1330909>

Macy, R. J., Jones, E., Graham, L. M., & Roach, L. (2018). Yoga for Trauma and Related Mental Health Problems: A Meta-Review With Clinical and Service Recommendations. *Trauma Violence Abuse*, *19*(1), 35-57. <https://doi.org/10.1177/1524838015620834>

Mandlik, G. V., Siopis, G., Nguyen, B., Ding, D., & Edwards, K. M. (2024). Effect of a single session of yoga and meditation on stress reactivity: A systematic review. *Stress Health*, *40*(3), e3324. <https://doi.org/10.1002/smi.3324>

Marklund, L., Wahlroos, T., Looi, G. E., & Gabrielsson, S. (2020). 'I know what I need to recover': Patients' experiences and perceptions of forensic psychiatric inpatient care. *International Journal of Mental Health Nursing*, *29*(2), 235-243. <https://doi.org/10.1111/inm.12667>

Mittelmark, M., Daniel, M., & Urke, H. (2022). Specific Resistance Resources in the Salutogenic Model of Health. In M. Mittelmark, G. Bauer, & L. e. a. Vaandrager (Eds.), *The Handbook of Salutogenesis* (2nd ed., pp. 107-114). Springer.

Olsson, A., Hedlund, S., & Landgren, K. (2022). To Use or Not Use Complementary and Alternative Medicine (CAM) in Psychiatric Care: Interviews with Clinical Decision-Makers in Sweden. *Issues in Mental Health Nursing*, *43*(5), 463-472. <https://doi.org/10.1080/01612840.2021.1986759>

O'Shea, M., Capon, H., Evans, S., Agrawal, J., Melvin, G., O'Brien, J., & McIver, S. (2022). Integration of hatha yoga and evidence-based psychological treatments for common mental disorders: An evidence map. *Journal of clinical psychology*, *78*(9), 1671–1711. <https://doi.org/10.1002/jclp.23338>

Pascoe, M. C., & Bauer, I. E. (2015). A systematic review of randomised control trials on the effects of yoga on stress measures and mood. *Journal of Psychiatric Research*, *68*, 270-282. <https://doi.org/10.1016/j.jpsychires.2015.07.013>

Penman, S., Cohen, M., Stevens, P., & Jackson, S. (2012). Yoga in Australia: Results of a national survey. *Int J Yoga*, *5*(2), 92-101. <https://doi.org/10.4103/0973-6131.98217>

Polit, D. F., & Beck, C. T. (2021). *Nursing research: generating and assessing evidence for nursing practice*. Wolters Kluwer

Pollak, C., Palmstierna, T., Kald, M., & Ekstrand, P. (2018). "It Had Only Been a Matter of Time Before I Had Relapsed Into Crime": Aspects of Care and Personal Recovery in Forensic Mental Health. *Journal of Forensic Nursing*, *14*(4), 230-237. <https://doi.org/10.1097/jfn.0000000000000210>

Sathyanarayanan, G., Vengadavaradan, A., & Bharadwaj, B. (2019). Role of Yoga and Mindfulness in Severe Mental Illnesses: A Narrative Review. *Int J Yoga*, *12*(1), 3-28. <https://doi.org/10.4103/ijoy.IJOY_65_17>

SFS 1991:1129. *Lag om rättspsykiatrisk vård*. <https://www.riksdagen.se/sv/dokument-och-lagar/dokument/svensk-forfattningssamling/lag-19911129-om-rattspsykiatrisk-vard_sfs-1991-1129/>

SFS 2003:460. *Lag om etikprövning av forskning som aver männsikor.* <https://www.riksdagen.se/sv/dokument-och-lagar/dokument/svensk-forfattningssamling/lag-2003460-om-etikprovning-av-forskning-som_sfs-2003-460/>

Singh, B., Olds, T., Curtis, R., Dumuid, D., Virgara, R., Watson, A., Szeto, K., Connor, E., Ferguson, T., Eglitis, E., Miatke, A., Simpson, C. E. M., & Maher, C. (2023). Effectiveness of physical activity interventions for improving depression, anxiety and distress: an overview of systematic reviews. *British Journal of Sports Medicine*, *57*(18), 1203. <https://doi.org/10.1136/bjsports-2022-106195>

Sistig, B., Friedman, S. H., McKenna, B., & Consedine, N. S. (2015). Mindful yoga as an adjunct treatment for forensic inpatients: a preliminary evaluation. *Journal of Forensic Psychiatry & Psychology*, *26*(6), 824-846. <https://doi.org/10.1080/14789949.2015.1062996>

Snaith, N., Rasmussen, P., Schultz, T., & Proeve, M. (2020). The practicability and relevance of developing a yoga intervention for mental health consumers: A qualitative study. *International Journal of Mental Health Nursing*, *29*(4), 622-631. <https://doi.org/10.1111/inm.12694>

Solomon, D., & Adams, J. (2015). The use of complementary and alternative medicine in adults with depressive disorders. A critical integrative review. *Journal of Affective Disorders*, *179*, 101-113. <https://doi.org/10.1016/j.jad.2015.03.031>

SOU 2019:28*. Komplementär och alternativ medicin och vård*. (Complementary and alternative medicine and care). <https://www.riksdagen.se/sv/dokument-och-lagar/dokument/statens-offentliga-utredningar/komplementar-och-alternativ-medicin-och-vard-_h7b328/html/>

Spinelli, C., Paradis-Gagné, E., Per, M., Fleischmann, M. H., Manova, V., Wallace, A., & Khoury, B. (2020). Evaluating the Use of Mindfulness and Yoga Training on Forensic Inpatients: A Pilot Study. *Front Psychiatry*, *11*, 614409. <https://doi.org/10.3389/fpsyt.2020.614409>

Suominen, S., & Lindstrom, B. (2008). Salutogenesis. *Scand J Public Health*, *36*(4), 337-339. <https://doi.org/10.1177/1403494808093268>

Tibbitts, DC., Aicher, SA., Sugg, J., Handloser, K., Eisman, L., Booth, LD. & Bradley, RD. (2021). Program evaluation of trauma-informed yoga for vulnerable populations. *Evaluation and Program Planning, 88,* Article 101946. doi: [10.1016/j.evalprogplan.2021.101946](https://doi.org/10.1016/j.evalprogplan.2021.101946)

Tong, A., Sainsbury, P., & Craig, J. (2007). Consolidated criteria for reporting qualitative research (COREQ): a 32-item checklist for interviews and focus groups. *International journal for quality in health care*, *19*(6), 349-357. <https://doi.org/10.1093/intqhc/mzm042>

Uebelacker, L. A., & Broughton, M. K. (2018). Yoga for Depression and Anxiety: A Review of Published Research and Implications for Healthcare Providers. *Focus (American Psychiatric Publishing)*, *16*(1), 95–97. <https://doi.org/10.1176/appi.focus.16104>

van der Kolk, B. A. (2014). *The body keeps the score: Brain, mind, and body in the healing of trauma.* Viking.

van der Kolk, B. A., Stone, L., West, J., Rhodes, A., Emerson, D., Suvak, M., & Spinazzola, J. (2014). Yoga as an adjunctive treatment for posttraumatic stress disorder: a randomized controlled trial. *Journal of Clinical Psychiatry*, *75*(6), e559-565. <https://doi.org/10.4088/JCP.13m08561>

Vancampfort, D., Stubbs, B., Van Damme, T., Smith, L., Hallgren, M., Schuch, F., Deenik, J., Rosenbaum, S., Ashdown-Franks, G., Mugisha, J., & Firth, J. (2021). The efficacy of meditation-based mind-body interventions for mental disorders: A meta-review of 17 meta-analyses of randomized controlled trials. *Journal of Psychiatric Research*, *134*, 181-191. <https://doi.org/10.1016/j.jpsychires.2020.12.048>

Vella, S. A., Aidman, E., Teychenne, M., Smith, J. J., Swann, C., Rosenbaum, S., White, R. L., & Lubans, D. R. (2023). Optimising the effects of physical activity on mental health and wellbeing: A joint consensus statement from Sports Medicine Australia and the Australian Psychological Society. *Journal of Science and Medicine in Sport*, *26*(2), 132-139. <https://doi.org/https://doi.org/10.1016/j.jsams.2023.01.001>

Wang, F., & Szabo, A. (2020). Effects of Yoga on Stress Among Healthy Adults: A Systematic Review. *Alternative Therapies in Health and Medicine*, *26*(4).

Wemrell, M., Olsson, A., & Landgren, K. (2020). The Use of Complementary and Alternative Medicine (CAM) in Psychiatric Units in Sweden. *Issues in Mental Health Nursing*, *41*(10), 946-957. <https://doi.org/10.1080/01612840.2020.1744203>

WHO. (1986). *The Ottawa charter for health promotion. First international conference on Health promotion*. <http://www.who.int/healthpromotion/conferences/previous/ottawa/en/>

World Medical Association. (2024). *WMA Declaration of Helsinki - Ethical principles for medical research involving human participants*. <https://www.wma.net/policies-post/wma-declaration-of-helsinki/>

Wu, Y., Yan, D., & Yang, J. (2023). Effectiveness of yoga for major depressive disorder: A systematic review and meta-analysis. *Front Psychiatry*, *14*, 1138205. <https://doi.org/10.3389/fpsyt.2023.1138205>

Yin, J., Sun, Y., Zhu, Y., Alifujiang, H., Wang, Y., An, S., Huang, H., Fu, X., Deng, H., & Chen, Y. (2024). Effects of yoga on clinical symptoms, quality of life and social functioning in patients with schizophrenia: A systematic review and meta-analysis. *Asian Journal of Psychiatry*, *93*, 103959. <https://doi.org/https://doi.org/10.1016/j.ajp.2024.103959>

Zaccari, B., Higgins, M., Haywood, T. N., Patel, M., Emerson, D., Hubbard, K., Loftis, J. M., & Kelly, U. A. (2023). Yoga vs Cognitive Processing Therapy for Military Sexual Trauma-Related Posttraumatic Stress Disorder: A Randomized Clinical Trial. *JAMA network open*, *6*(12), e2344862. <https://doi.org/10.1001/jamanetworkopen.2023.44862>

Zhong, S., Guo, H., Wang, Y., Cook, S., Chen, Y., Luo, C., Peng, K., Wang, F., Liang, X., Chen, H., Li, Q., Zhou, J., Wang, X., & Chen, R. (2019). The experience of long-stay patients in a forensic psychiatric hospital in China: a qualitative study. *BMC Health Services Research*, *19*(1), 617. <https://doi.org/10.1186/s12913-019-4458-6>
